# Supplementary material for: MDC1 PST-repeat region promotes histone H2AX-independent chromatin association and DNA damage tolerance
Source: Nat Commun. 2019 Nov 15;10:5191. doi: 10.1038/s41467-019-12929-5 (PMC6858307; doi:10.1038/s41467-019-12929-5)
Supplement: Supplementary file 1 — Supplementary Information [file 41467_2019_12929_MOESM1_ESM.pdf]

MDC1 PST-repeat region promotes histone H2AX-independent chromatin association and DNA damage tolerance

Salguero et al.

Supplementary Materials including:

Supplementary Tables 1 - 4

Supplementary Figures 1 - 6

Targeted gene	Oligonucleotide name	Oligonucleotide sequence
H2AFX	H2AFX FSgRNA1 Fwd	ACCGTACGCCGAGCGCGTTGGCGC
	H2AFX FSgRNA1 Rev	AAACGCGCCAACGCGCTCGGCGTA
	H2AFX RSgRNA1 Fwd	ACCGTCCGCAGCAGCCGGTGTACA
	H2AFX RSgRNA1 Rev	AAACTGTACACCGGCTGCTGCGGA

Supplementary Table 1. Sequences of DNA templates used for sgRNA production.

Gene	Oligonucleotide name	Oligonucleotide sequence
H2AFX	H2AFX Fwd	CCCATCAGACCCCGTCTAAC
	H2AFX Rev	CCCCTCCCACCCCTATTATCA
	H2AX seq1 Fw	GGATAGTTGGCAGTCTGCGT
	H2AX seq1 Rv	AGCTTGTTGAGCTCCTCGTC
EGFP	EGFP-C	CATGGTCCTGCTGGAGTTCGTG
MDC1	PST_r2_R	TATACCCGGGCTGGAGCTCAAGGGCTGTGG
	PST_r6_R	TATACCCGGGTGGGAGCTCAGGGGCTATAG

Supplementary Table 2. Sequences of DNA primers used in this work.

Gene	Sequence
53BP1	GAAGGACGGAGUACUAAUA
RNF8	UGGACAAUUAUGGACAACA +<br>GCACAGAAGGAAGAAGUUC
SHLD1	GCUUUCAGUUCUUUGGAAU
SHLD2	GCACCUUCAACCUGAUGUA
Luciferase	CGUACGCGGAAUACUUCGA

Supplementary Table 3. siRNA sequences used for protein depletion.

Antigen	Antibody	Application (dilution)	Origin specie
GFP	Roche 11814460001	WB 1:1000	Mouse
H2A	Abcam ab18255	WB 1:1000	Rabbit
MDC1	Bethyl A300-051A	WB 1:750	Rabbit
	Abcam ab11171	IF 1:500	Rabbit
H2AXpS139	Millipore 05-636	WB 1:1000	Mouse
		IF 1:1000	
H2AX	Abcam ab11175	WB 1:5000	Rabbit
DNA-PKcs-pS2056	Abcam ab18192	WB 1:200	Rabbit
DNA-PKcs	Serotec AHP318	WB 1:2000	Rabbit
KAP1-pS824	Bethyl IHC-00073	WB 1:1000	Rabbit
KAP1	Abcam ab10483	WB 1:10000	Rabbit
CHK2-pT68	Cell Signalling 2661S	WB 1:1000	Rabbit
CHK2	Abcam ab8108	WB 1:1000	Rabbit
H2B	Abcam ab1790	WB 1:5000	Rabbit
H3	Abcam ab1791	WB 1:100000	Rabbit
H4	Abcam ab10158	WB 1:10000	Rabbit
HA	Santa Cruz sc7392	WB 1:500	Mouse
RAD50	Serotec AHP798	WB 1:2000	Sheep
53BP1	Novus Biologicals NB100-304	IF 1:1000	Rabbit
		WB 1:5000	
GAPDH	Millipore MAB374	WB 1:1000	Mouse
Cyclin A	BD Bioscience 611268	IF 1:250	Mouse
α-Tubulin	Sigma-Aldrich T9026	WB 1:50000	Mouse
PARP1	Cell Signalling 9542	WB 1:2000	Rabbit

Supplementary Table 4. Antibodies used in this work.
WB: western blotting; FC: flow cytometry; IF: immunofluorescence.

a

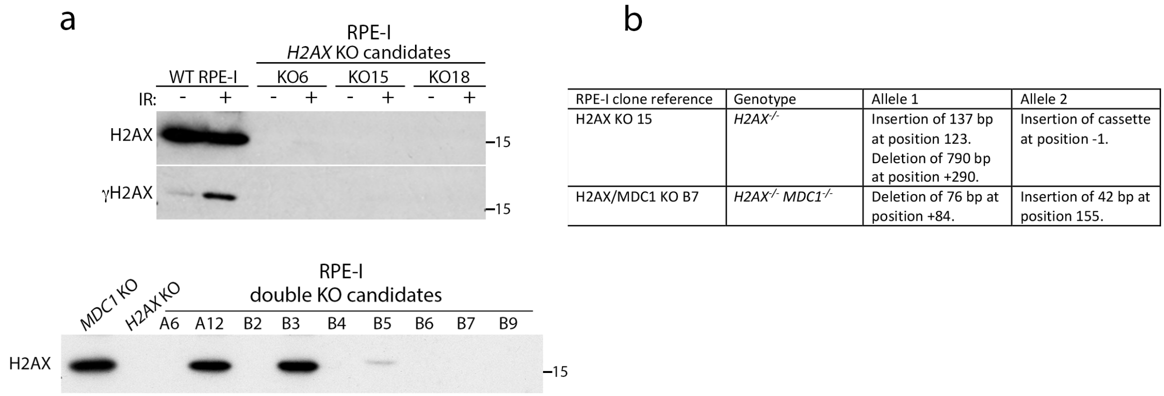

b

RPE-I clone reference	Genotype	Allele 1	Allele 2
H2AX KO 15	*H2AX$^{-/-}$*	Insertion of 137 bp at position 123. Deletion of 790 bp at position +290.	Insertion of cassette at position -1.
H2AX/MDC1 KO B7	*H2AX$^{-/-}$ MDC1$^{-/-}$*	Deletion of 76 bp at position +84.	Insertion of 42 bp at position 155.

c

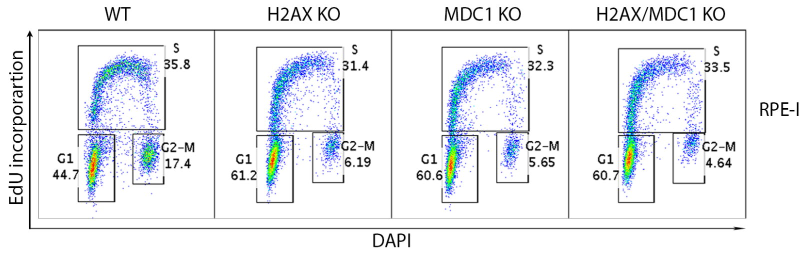

d

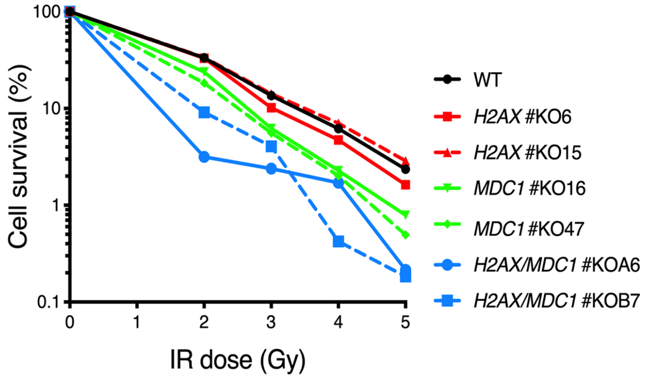

e

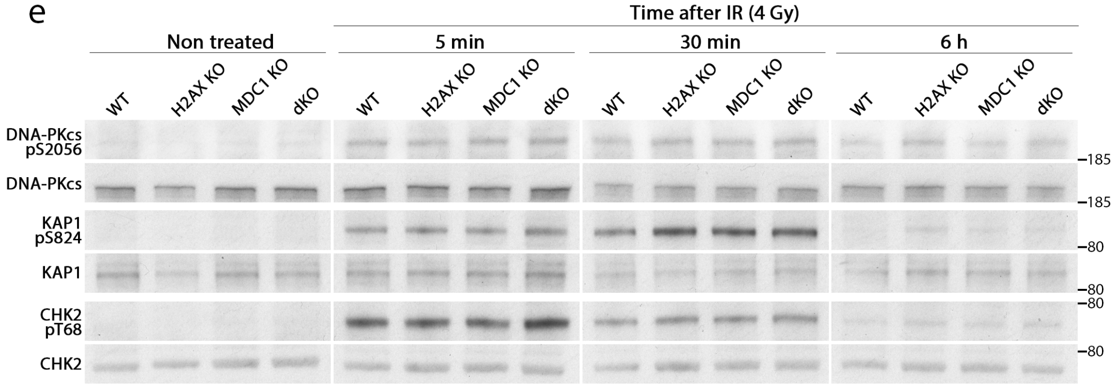

Supplementary figure 1. (a) Verification western blots for knocking out of *H2AX* in wild-type (top panel) and in *MDC1*$^{-/-}$ (bottom panel) hTERT RPE-1 cells. *MDC1*$^{-/-}$ hTERT RPE-1 cells were generated previously[1]. **(b)** Genotypes of the RPE-1 knockout clones used in this work confirmed by Topo-cloning and Sanger sequencing. **(c)** Cell cycle profiles assessed by EdU incorporation and DAPI of the RPE-1 knockout cells used in this work. **(d)** Clonogenic survival assay after treating two clones for each knockout background with the indicated IR doses. **(e)** Representative western blot images showing phosphorylations of the indicated proteins involved in DSB signaling pathway and checkpoint activation after IR treatment of wild-type RPE-1 (WT) and indicated mutant cell lines.

Salguero et al. Supplementary Figure 2

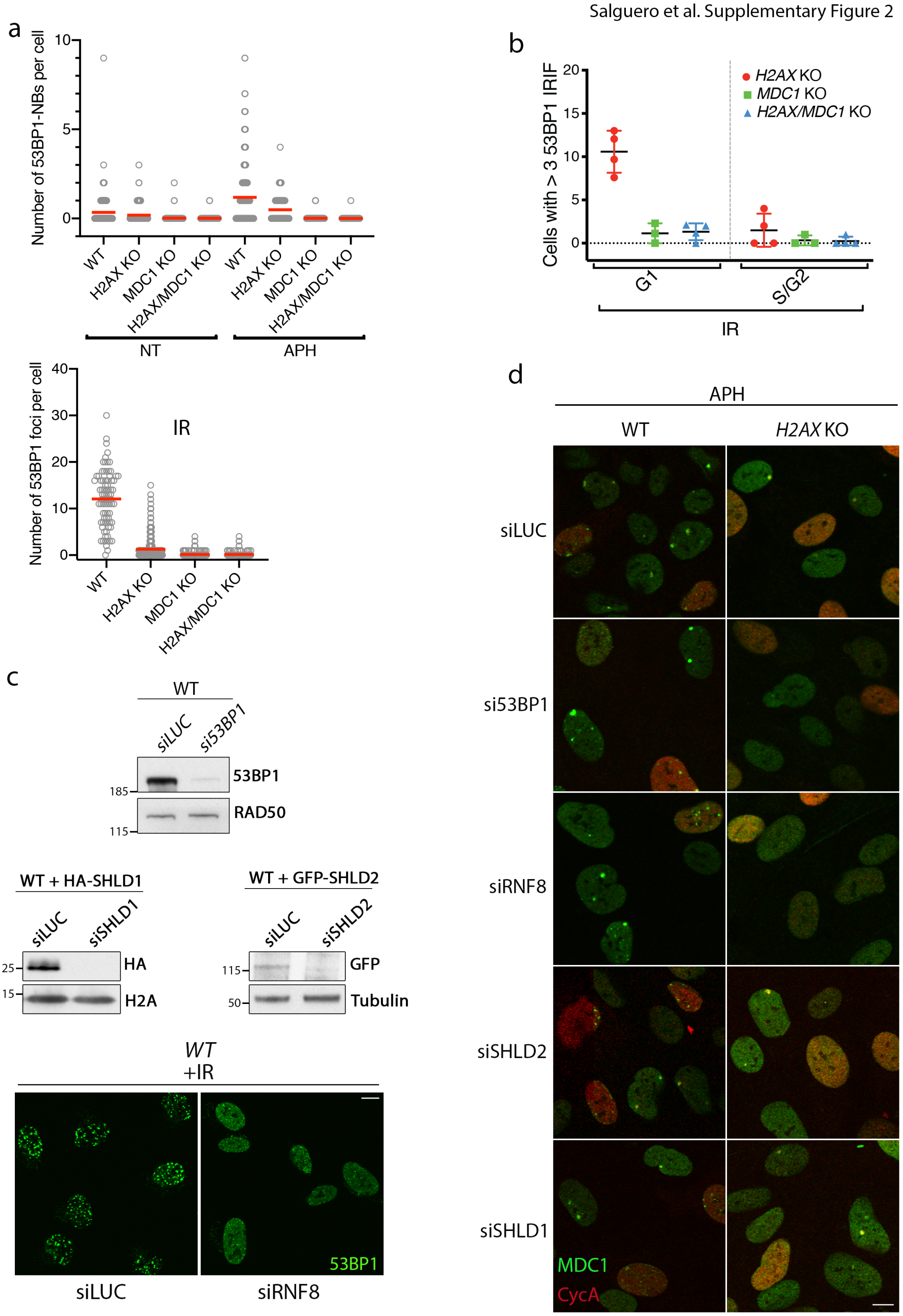

Supplementary figure 2. (a) Top panel: quantification of number of 53BP1-NBs per G1 cell after APH treatment from the experiment corresponding to figure 2a and b. Bottom panel: quantification of 53BP1 foci per cell after IR treatment from the experiment corresponding to figure 2a and b. Red bar mean. **(b)** Quantification of number of IRIF-positive RPE-1 cells from experiment corresponding to figure 2a and b depending of their cell-cycle stage (determined by Cyclin A staining); n = 4/genotype (except for *MDC1* KO n = 3); error bars s.e.m. **(c)** Top panels: Western blots to verify depletion of 53BP1, SHLD1 and SHLD2 in RPE-1 cells 48h after transfection with siRNAs. Due to the lack of good antibodies to detect endogenous SHLD1 and SHLD2, the efficiency of these siRNAs was tested on cells transfected with plasmids expressing HA-SHLD1 or GFP-SHLD2[2]. Bottom panel: due to the lack of a good antibody to directly verify RNF8 depletion, 53BP1 IRIF formation was used as a readout of RNF8 activity. Cells were irradiated (3 Gy) 48h after transfection with RNF8 siRNA. **(d)** Representative immunofluorescence images showing MDC1 localization at NBs after 24h treatment with 0.4 μM APH. RPE-1 cells were depleted of 53BP1, RNF8, SHLD1 or SHLD2 by siRNA for 48h before APH treatment. Scale bars, 10 μm.

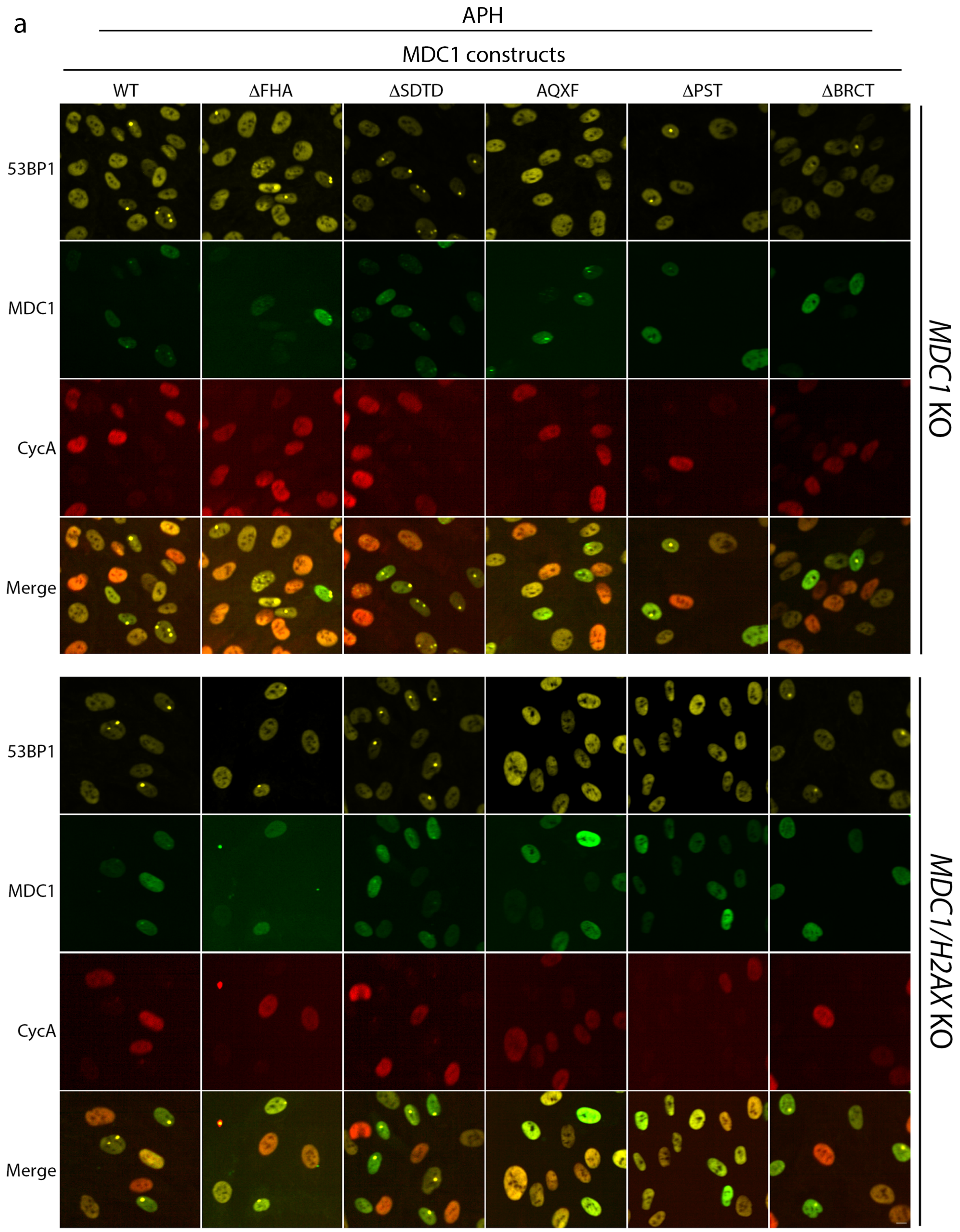
a
APH
MDC1 constructs
WT
ΔFHA
ΔSDTD
AQXF
ΔPST
ΔBRCT
53BP1
MDC1
CycA
Merge
MDC1 KO
53BP1
MDC1
CycA
Merge
MDC1/H2AX KO

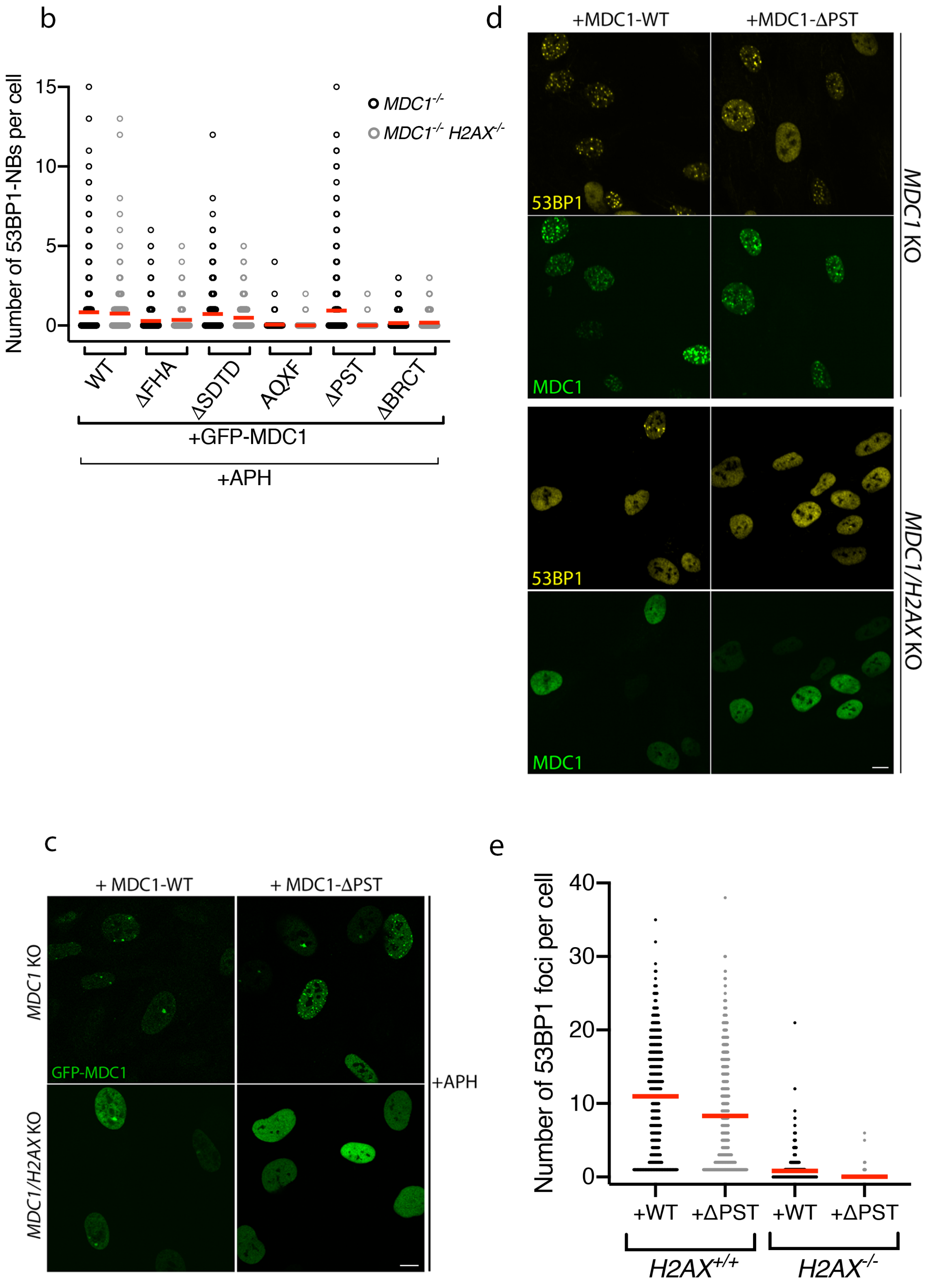
b
Number of 53BP1-NBs per cell
15
10
5
0
MDC1-/-
MDC1-/- H2AX-/-
WT
ΔFHA
ΔSDTD
AQXF
ΔPST
ΔBRCT
+GFP-MDC1
+APH
d
+MDC1-WT
+MDC1-ΔPST
53BP1
MDC1
MDC1 KO
53BP1
MDC1
MDC1/H2AX KO
c
+ MDC1-WT
+ MDC1-ΔPST
MDC1 KO
GFP-MDC1
MDC1/H2AX KO
+APH
e
Number of 53BP1 foci per cell
40
30
20
10
0
+WT
+ΔPST
+WT
+ΔPST
H2AX+/+
H2AX-/-

Supplementary figure 3. (a) Representative images of RPE-1 *MDC1*$^{-/-}$ *H2AX*$^{+/+}$ and *MDC1*$^{-/-}$ *H2AX*$^{-/-}$ cells complemented with indicated WT or mutant versions of GFP-MDC1 treated with 0.4 μM APH for 24h that were used for quantifications shown in figure 3b. **(b)** Quantification of number of 53BP1-NBs per G1 cell after APH treatment from images corresponding to the experiment shown in figure 3b and supplementary figure 3a**.** Red bar mean**. (c)** Representative immunofluorescence images of MDC1-NB formation after APH treatment (as in a) in RPE-1 *MDC1*$^{-/-}$ *H2AX*$^{+/+}$ and *MDC1*$^{-/-}$ *H2AX*$^{-/-}$ cells complemented either with WT or ΔPST versions of GFP-MDC1. **(d)** Representative immunofluorescence images of MDC1 and 53BP1 IRIF 1h after IR (3 Gy) treatment of the cells complemented either with WT or ΔPST versions of GFP-MDC1. **(e)** Quantification of 53BP1 foci per cell after IR treatment from images corresponding to the experiment shown in figure 3d and supplementary figure 3d. Red bar mean. Scale bars, 10 μm.

a

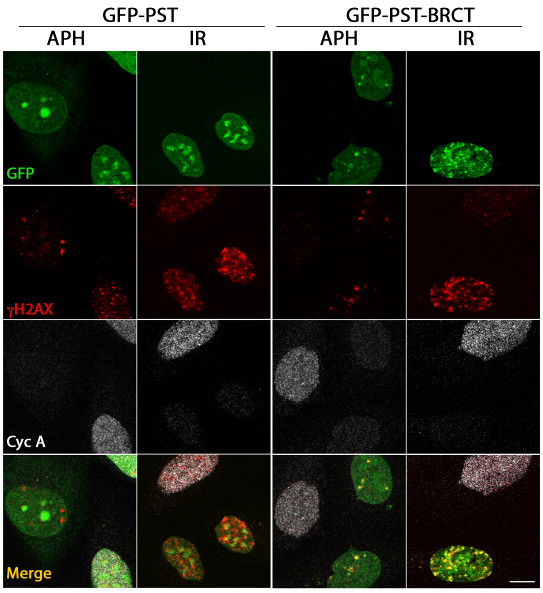

b

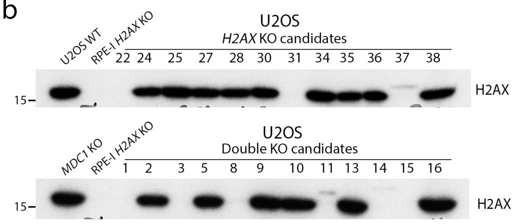

c

U2OS clone reference	Genotype	Allele 1	Allele 2
H2AX KO 22	*H2AX*$^{-/-}$	Deletion of 27 bp at position 104.	Deletion of 75 bp at position 44.
H2AX/MDC1 KO 15	*H2AX*$^{-/-}$ *MDC1*$^{-/-}$	Deletion of 1325 bp at position 43.	Deletion of 1325 bp at position 43.

d

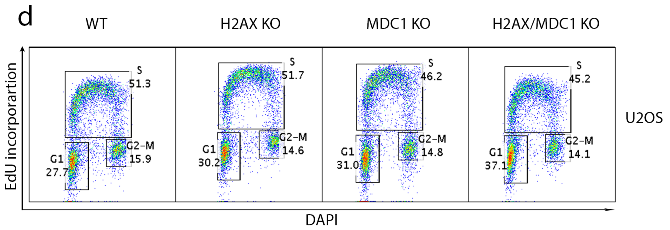

e

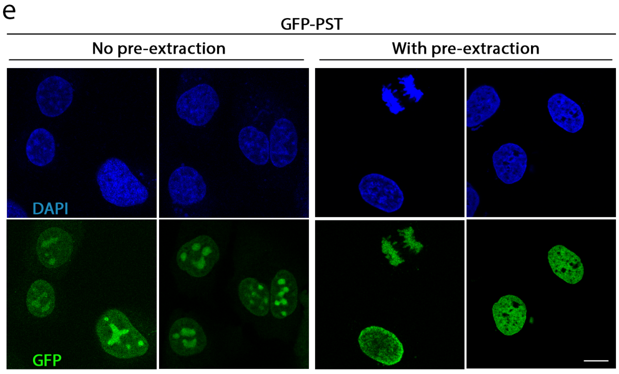

f

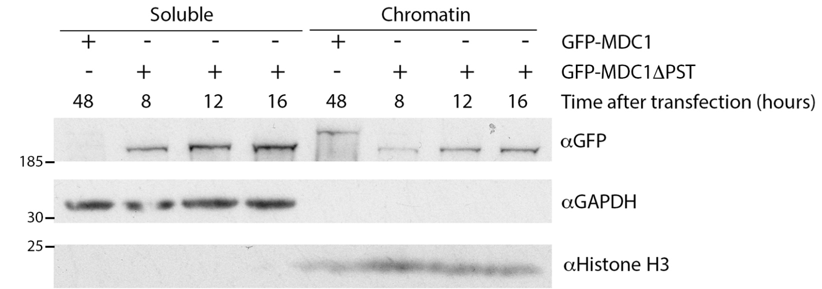
Soluble
Chromatin
GFP-MDC1
GFP-MDC1ΔPST
Time after transfection (hours)
48
8
12
16
48
8
12
16
αGFP
αGAPDH
αHistone H3
185
30
25

g

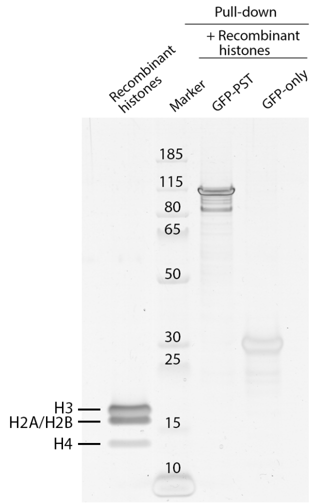
Pull-down
+ Recombinant histones
Recombinant histones
Marker
GFP-PST
GFP-only
185
115
80
65
50
30
25
15
10
H3
H2A/H2B
H4

h

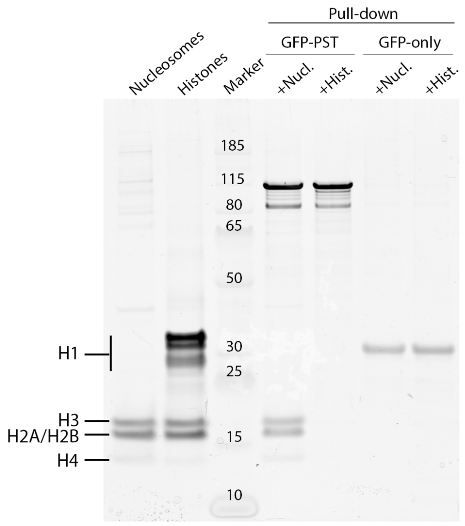
Pull-down
GFP-PST
GFP-only
Nucleosomes
Histones
Marker
+Nucl.
+Hist.
+Nucl.
+Hist.
185
115
80
65
50
30
25
15
10
H1
H3
H2A/H2B
H4

i

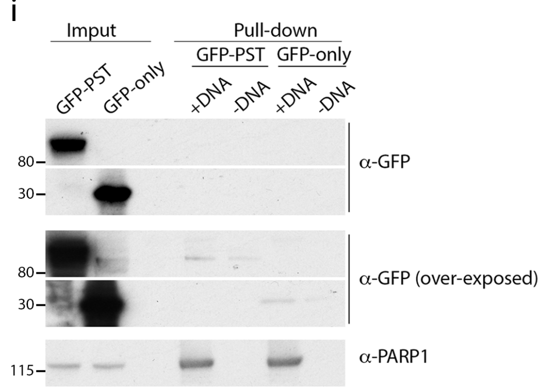
Imput
Pull-down
GFP-PST
GFP-only
GFP-PST
GFP-only
+DNA
-DNA
+DNA
-DNA
80
30
α-GFP
80
30
α-GFP (over-exposed)
115
α-PARP1

Supplementary figure 4. (a) Representative immunofluorescence images of U2OS cells transfected with plasmids expressing GFP-PST or GFP-PST-BRCT and treated with 0.4 μM APH for 24h or fixed 1h after IR (3 Gy). **(b)** Verification western blots for knocking out of *H2AX* in wild-type (top panel) or *MDC1*$^{-/-}$ (bottom panel) U2OS cells. *MDC1*$^{-/-}$ U2OS cells were generated previously[1]. **(c)** Genotypes of the U2OS knockout clones used in this work confirmed by Topo-cloning and Sanger sequencing. **(d)** Cell cycle profiles assessed by EdU incorporation and DAPI of the U2OS knockout cells used in this work. **(e)** Representative immunofluorescence images of U2OS cells expressing GFP-PST with or without pre-extraction before fixation. **(f)** Chromatin fractionation of U2OS *MDC1*$^{-/-}$ *H2AX*$^{-/-}$ cells at indicated times after transfection with *GFP-MDC1* or *GFP-MDC1ΔPST*. **(g)** Representative silver staining image of biochemical protein binding assays between GFP-PST or GFP-only, purified from HEK293 cells, and a commercial cocktail of recombinant core histones. **(h)** Representative silver staining image of biochemical protein binding assay between purified GFP-PST or GFP-only and histones purified from calf thymus or mono-nucleosomes purified from HeLa cells. **(i)** DNA binding assay of GFP-PST and GFP-only purified from HEK293 cells. Scale bars, 10 μm.

Salguero et al. Supplementary Figure 5

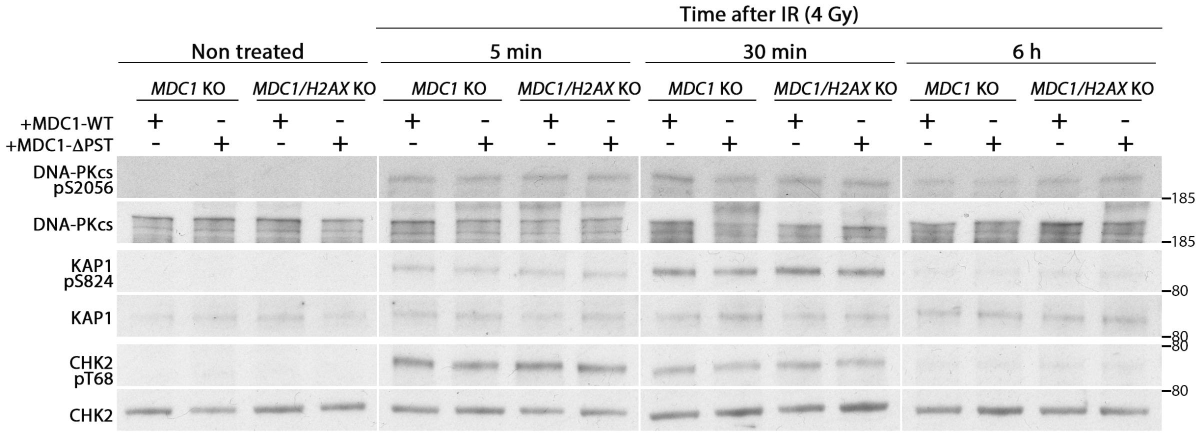

Supplementary figure 5. Representative western blot images showing the indicated proteins and phosphorylations after IR treatment of RPE-1 *MDC1*$^{-/-}$ and the *MDC1*$^{-/-}$ *H2AX*$^{-/-}$ mutant cell lines complemented with GFP-MDC1 or GFP-MDC1ΔPST constructs.

Salguero et al. Supplementary Figure 6a

Related to figure 4a

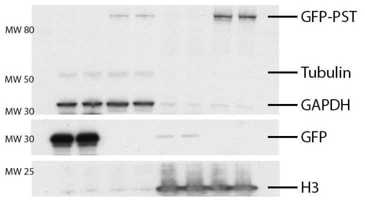

Related to figure 4b

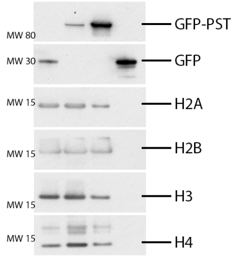

Related to figure 4c

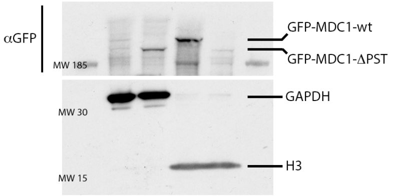

Related to figure 4d

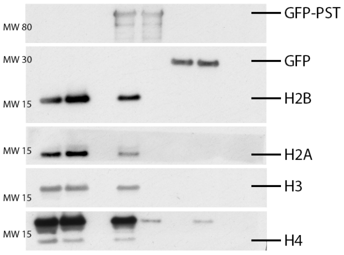

Related to figure 5b

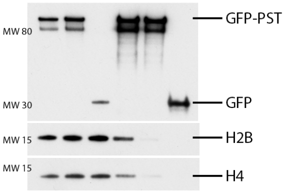

Related to figure 5c

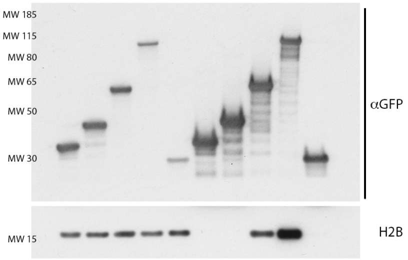

Related to figure 5d

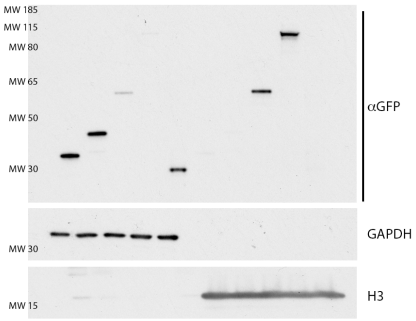

Related to Supplementary Figure 1a (Top panel)

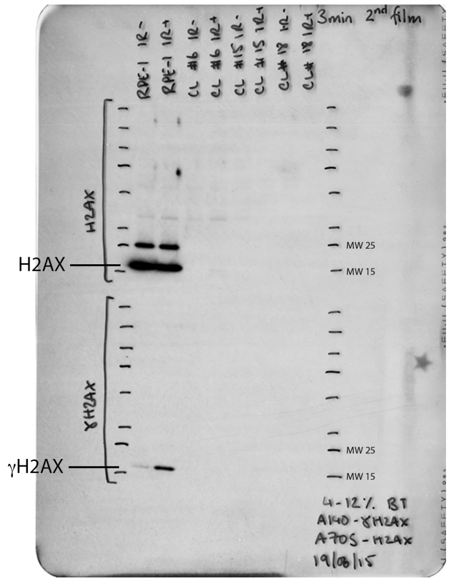

Related to Supplementary Figure 1e

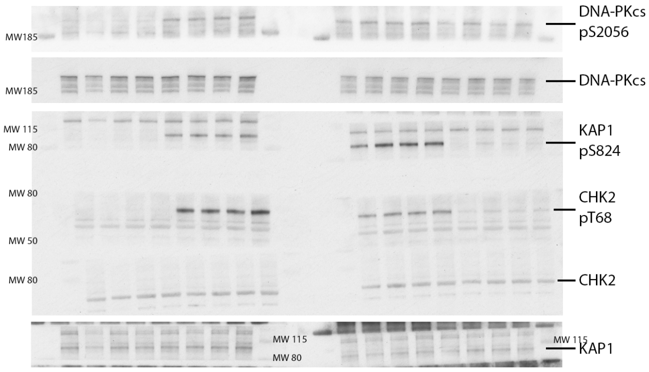

Related to Supplementary Figure 1a (Bottom panel)

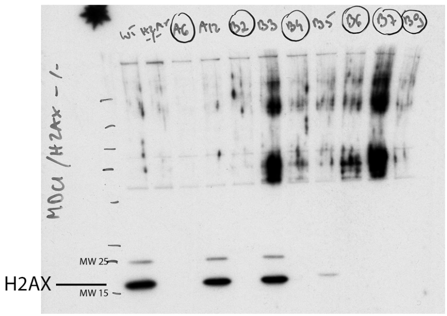

Related to Supplementary Figure 2c

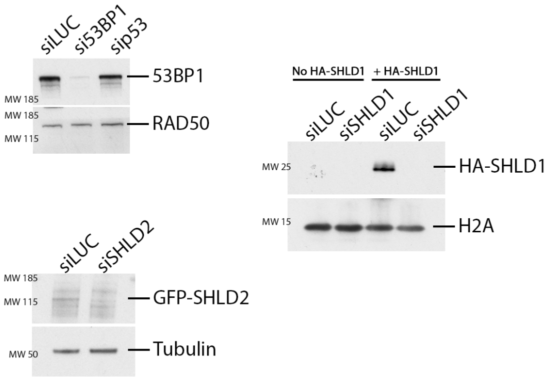

Related to Supplenetary Figure 4b (Bottom panel)

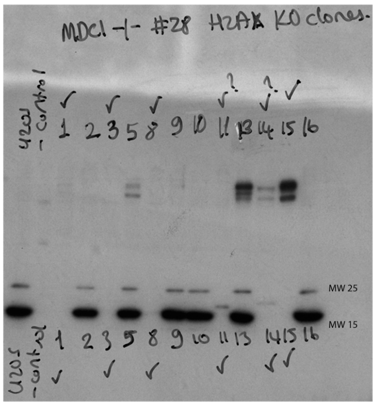

Related to Supplementary Figure 4b (Top panel)

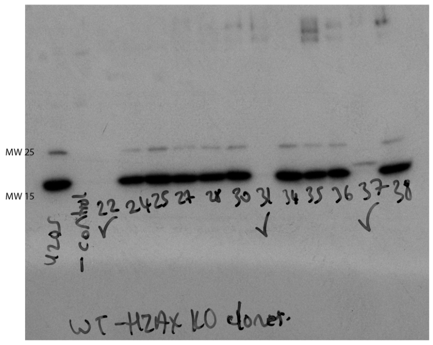

Related to Supplementary Figure 4f

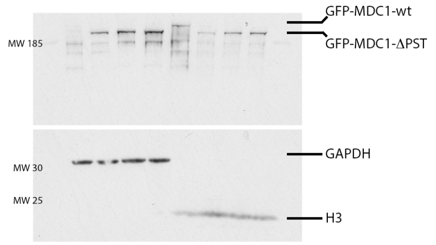

Related to Supplementary Figure 4i

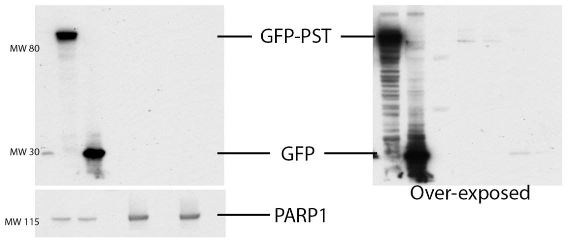

Related to Supplementary Figure 5

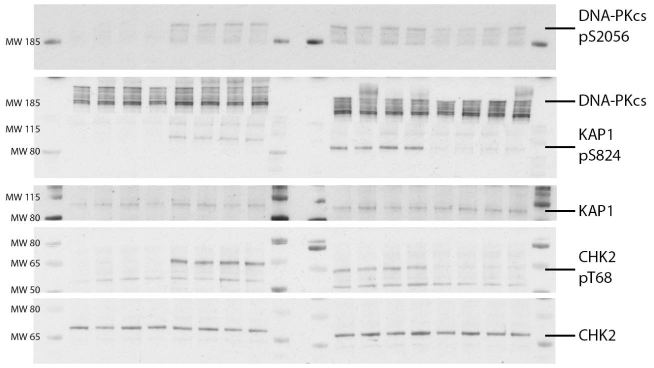

Supplementary figure 6 (a and b). Image scans for all the western blot data used in the figures of the present manuscript.

Supplementary references

1 Chiang, T. W., le Sage, C., Larrieu, D., Demir, M. & Jackson, S. P. CRISPR-Cas9(D10A) nickase-based genotypic and phenotypic screening to enhance genome editing. *Sci Rep* **6**, 24356, doi:10.1038/srep24356 (2016).

2 Dev, H. *et al.* Shieldin complex promotes DNA end-joining and counters homologous recombination in BRCA1-null cells. *Nature Cell Biology*, doi:10.1038/s41556-018-0140-1 (2018).
