# Supplementary material for: MDC1 PST-repeat region promotes histone H2AX-independent chromatin association and DNA damage tolerance
Source: Nat Commun. 2019 Nov 15;10:5191. doi: 10.1038/s41467-019-12929-5 (PMC6858307; doi:10.1038/s41467-019-12929-5)
Supplement: Supplementary file 3 — Reporting Summary [file 41467_2019_12929_MOESM3_ESM.pdf]

nature research

Corresponding author(s): Stephen P. Jackson

Last updated by author(s): Sep 24, 2019

Reporting Summary

Nature Research wishes to improve the reproducibility of the work that we publish. This form provides structure for consistency and transparency in reporting. For further information on Nature Research policies, see Authors & Referees and the Editorial Policy Checklist.

Statistics

For all statistical analyses, confirm that the following items are present in the figure legend, table legend, main text, or Methods section.

n/a	Confirmed	
☐	☒	The exact sample size (*n*) for each experimental group/condition, given as a discrete number and unit of measurement
☐	☒	A statement on whether measurements were taken from distinct samples or whether the same sample was measured repeatedly
☐	☒	The statistical test(s) used AND whether they are one- or two-sided<br>*Only common tests should be described solely by name; describe more complex techniques in the Methods section.*
☐	☒	A description of all covariates tested
☐	☒	A description of any assumptions or corrections, such as tests of normality and adjustment for multiple comparisons
☐	☒	A full description of the statistical parameters including central tendency (e.g. means) or other basic estimates (e.g. regression coefficient) AND variation (e.g. standard deviation) or associated estimates of uncertainty (e.g. confidence intervals)
☐	☒	For null hypothesis testing, the test statistic (e.g. *F*, *t*, *r*) with confidence intervals, effect sizes, degrees of freedom and *P* value noted<br>*Give P values as exact values whenever suitable.*
☒	☐	For Bayesian analysis, information on the choice of priors and Markov chain Monte Carlo settings
☒	☐	For hierarchical and complex designs, identification of the appropriate level for tests and full reporting of outcomes
☒	☐	Estimates of effect sizes (e.g. Cohen's *d*, Pearson's *r*), indicating how they were calculated

Our web collection on statistics for biologists contains articles on many of the points above.

Software and code

Policy information about availability of computer code

Data collection	Acquisition of confocal immunofluorescence microscopy images was carried out by using the LAS X 3.5.2 software (Leica Microsystems). Harmony 4.8 software (PerkinElmer) was used for Automated high-throughput/high-content microscopy image acquisition. Additional software used in this study include Microsoft Excel for Mac (Microsoft Inc, USA). Adobe Photoshop and Illustrator were used to process data for publication (Adobe Systems Inc, USA).
Data analysis	Above statistical parameters are indicated in the methods section. Graph display and statistical analysis was performed using Prism v7 for MacOs (GraphPad Software Inc., USA). Confocal microscopy images were processed by ImageJ (version 2.0.0) and quantified by Volocity 6.3 (PerkinElmer). For the Automated high-throughput/high-content microscopy images, the different quantifications were carried out by using Harmony 4.8 software (PerkinElmer).

For manuscripts utilizing custom algorithms or software that are central to the research but not yet described in published literature, software must be made available to editors/reviewers. We strongly encourage code deposition in a community repository (e.g. GitHub). See the Nature Research guidelines for submitting code & software for further information.

Data

Policy information about availability of data

All manuscripts must include a data availability statement. This statement should provide the following information, where applicable:
- Accession codes, unique identifiers, or web links for publicly available datasets
- A list of figures that have associated raw data
- A description of any restrictions on data availability

All the data and materials supporting this work are available upon reasonable request to the corresponding author.

Field-specific reporting

Please select the one below that is the best fit for your research. If you are not sure, read the appropriate sections before making your selection.

☒ Life sciences ☐ Behavioural & social sciences ☐ Ecological, evolutionary & environmental sciences

For a reference copy of the document with all sections, see nature.com/documents/nr-reporting-summary-flat.pdf

Life sciences study design

All studies must disclose on these points even when the disclosure is negative.

Sample size	All experiments were conducted with cell lines with multiple available biological replicates and based on previous experience with specific experimental setup; no statistical method was used to determine sample size.
Data exclusions	No data was excluded from analysis in this study.
Replication	All experiments were reliably reproduced as stated in the text, and detailed methods provided to aid in their replication by others. Where further methods/data are sought corresponding authors will oblige reasonable requests.
Randomization	No randomization method was used.
Blinding	Investigators were not blinded to group allocation during data collection and analysis. However, quantification of the number of foci per nucleus was performed in an unbiased way with the automated image-analysis software Harmony 4.8 or by Volocity 6.3 (both of them by PerkinElmer).

Reporting for specific materials, systems and methods

We require information from authors about some types of materials, experimental systems and methods used in many studies. Here, indicate whether each material, system or method listed is relevant to your study. If you are not sure if a list item applies to your research, read the appropriate section before selecting a response.

Materials & experimental systems

n/a	Involved in the study
☐	☒ Antibodies
☐	☒ Eukaryotic cell lines
☒	☐ Palaeontology
☒	☐ Animals and other organisms
☒	☐ Human research participants
☒	☐ Clinical data

Methods

n/a	Involved in the study
☒	☐ ChIP-seq
☐	☒ Flow cytometry
☒	☐ MRI-based neuroimaging

Antibodies

Antibodies used	Antibodies used in this study are listed in the "Materials and Methods" section of the manuscript.
Validation	Commercially available antibodies were validated by the supplier and by us using appropriate controls where needed.

Eukaryotic cell lines

Policy information about cell lines

Cell line source(s)	RPE1, HEK293, U2OS: S.P. Jackson Lab (Gurdon Institute, Cambridge, UK).
Authentication	All cells were originally obtained from the ATCC cell repository, and we have authenticated cell lines used in our study by STR profiling.
Mycoplasma contamination	All cells are routinely tested to be mycoplasma free.
Commonly misidentified lines (See ICLAC register)	No commonly misidentified cell lines were used.

Flow Cytometry

Plots

Confirm that:

☒ The axis labels state the marker and fluorochrome used (e.g. CD4-FITC).

☒ The axis scales are clearly visible. Include numbers along axes only for bottom left plot of group (a 'group' is an analysis of identical markers).

☒ All plots are contour plots with outliers or pseudocolor plots.

☒ A numerical value for number of cells or percentage (with statistics) is provided.

Methodology

Sample preparation	A full description of the sample preparation is detailed in the Methods section.
Instrument	Attune NxT (Invitrogen).
Software	The data was collected and analysed using FlowJo (BD Inc, USA).
Cell population abundance	*Describe the abundance of the relevant cell populations within post-sort fractions, providing details on the purity of the samples and how it was determined.*
Gating strategy	Doublets were distinguished from single cells by plotting the DAPI fluorescence (height vs area).

☐ Tick this box to confirm that a figure exemplifying the gating strategy is provided in the Supplementary Information.
